# Extracellular Vesicles in Lung Cancer: Bystanders or Main Characters?

**DOI:** 10.3390/biology12020246

**Published:** 2023-02-03

**Authors:** Mariaenrica Tinè, Davide Biondini, Marco Damin, Umberto Semenzato, Erica Bazzan, Graziella Turato

**Affiliations:** Department of Cardiac, Thoracic, Vascular Sciences and Public Health, University of Padova, 35128 Padova, Italy

**Keywords:** EV biomarkers, bronchoalveolar lavage, pleural fluid

## Abstract

**Simple Summary:**

Lung cancer is the biggest killer among cancers. Its mortality is related to delayed diagnosis which is often achieved in the advanced stages of the disease when surgery is no longer an option. Innovative treatments are now available for patients carrying specific genetic or immunological signatures. Tissue biopsy represents the gold standard sample to identify such life-changing therapeutic targets but obtaining it is not always easy, is often risky, and requires accurate radiological and clinical preliminary study. In this scenario, extracellular vesicles (EV), submicron particles released by both tumor and host cells, have been shown to carry strategical effectors and modulators of cancer development and to realize intercellular signaling. EV have been applied as a biosensor of disease showing high performance in both diagnosis and screening of lung cancer. As reviewed herein, circulating EV and especially those detected in body fluids in direct contact with lung cancer—bronchoalveolar lavage and pleural fluids—might convey not only tumor features but also key drivers of disease.

**Abstract:**

Lung cancer still represents the main cause of cancer death worldwide. The poor survival is mainly related to the diagnosis which is often obtained in advanced stages when the disease is unresectable and characterized by the worst prognosis. Only in the last decades have great discoveries led to the development of new therapies targeted to oncogenes and to boost the host immune response against the tumor. Tumor identification and molecular/immunological characterization rely on bioptic samples which represent the gold standard for diagnosis. Nonetheless, less invasive procedures providing small samples will be more and more common in the future. Extracellular vesicles (EV), submicron particles released by any cell type, are candidates for diagnostic and prognostic biomarkers. EV are mediators of intercellular communication and can convey cytokines, miRNAs, antigens, and many other factors of tumorigenesis. This review summarizes the most appealing findings on lung-cancer-related EV, debating the evidence on circulating versus airway EV as potential biomarkers in disease management and the main studies on the role of these particles on lung cancer pathogenesis. Overall, the available results point toward a wide range of possible applications, supported by the promising achievements of genotyping on BAL fluid EV and proteomic analysis on pleural effusion EV. Nonetheless, the study of lung EV is still affected by remarkable methodological issues, especially when in vitro evidence is translated into humans. Whether EV still represent an “information fog” or can be useful in lung cancer management will be discussed, with possible hints on how to improve their usage.

## 1. Introduction

Lung cancer still represents the main cause of cancer death worldwide, with an estimated 1.8 million deaths each year [1]. Primary lung cancer is typically divided into two main histopathological types: small-cell lung cancer (SCLC) and non-small-cell lung cancer (NSCLC). NSCLC accounts for 85% of lung cancer cases, including three main subtypes, namely adenocarcinoma, squamous cell carcinoma, and large cell carcinoma [2]. Despite the increased awareness of the risks related to tobacco smoking and a sharp reduction in smoking habits among men, in the last decades the number of smoking women has progressively increased. The new balance between genders and smokers/never smokers shifted the most prevalent lung cancer histotype from squamous cell carcinoma, typically affecting heavy smokers, toward adenocarcinoma which doubled its incidence from 1973 (8.9/100,000 person-years) to 2015 (20.3/100,000 person-years) [3]. The five-year relative survival rate for lung cancer has significantly increased over the years, mainly due to progress in treatment [3]. However, the survival of lung cancer patients is still poor, mainly because the disease is often diagnosed at an advanced stage when surgery, which offers the best chance of long-term survival, is not an option [4]. The identification of oncogene driver targets or of altered immune tumor environment now represents the goal of therapeutic strategies [4]. In this scenario, the appeal of rapidly and non-invasively assessable biomarkers for lung cancer screening and prognosis is unmeasurable. 

Extracellular vesicles (EV) are submicron particles released into biological fluids (including blood, saliva, urine, and cerebrospinal fluid) by every cell. EV are involved in cell-to-cell communication in both homeostasis and diseases, where they probably represent an alternative mechanism of intercellular signaling. Even though their nomenclature is still debated [5,6], the main EV subtypes investigated are exosomes (endosome-originated particles) and the larger plasma membrane-derived microvesicles (formerly called “microparticles”) [7].

Originally considered nothing more than “sticky cell debris”, their importance is rapidly growing as candidate biomarkers of several conditions and potential cargoes of therapeutic compounds [6]. Whether EV release is the result or the trigger of the disease, their “sense” is not fully elucidated. 

## 2. Circulating Extracellular Vesicles in Lung Cancer

EV are released by any cell type, tumor cell included, and share features of parental cells. In 2009, Rabinowits’s et al. observed that a) the miRNA content of circulating exosomes reflected tissue miRNA content and b) the total amount of circulating exosomes and the level of exosomal miRNA were much higher in patients with NSCLC than in controls [8]. Since then, several miRNAs have been associated with lung cancer screening and prognosis. By the assessment of a wide range of targets, O’Farrel et al. set up a signature of extravesicular miRNAs able to distinguish between healthy smokers and patients with lung cancer by a simple plasma collection [9]. This combination of circulating extravesicular miRNAs seems helpful to discriminate the nature of indeterminate lung nodules and categorize them as benign or malign [10]. Further evidence comes from a multicenter, prospective study that examined plasma samples from 460 subjects, including patients with early lung adenocarcinoma, benign pulmonary nodules, and healthy participants. This study validated an EV miRNA signature of 4 miRNA (hsa-miR-106b-3p, hsa-miR-125a-5p, hsa-miR-3615, and hsa-miR-450b-5p) that could distinguish early adenocarcinoma with a sensitivity of 83.8% and a specificity of 87.1% [11]. Another group [12] observed a significant and consistent increase in miR-520c-3p and miR-1274b in NSCLC patients compared to healthy controls and benign nodule patients (*p* < 0.001). These miRNAs markedly decreased after tumor resection (*p* < 0.001). In another study, EV let-7b-5p, miR-184 levels combined with peripheral miR-22-3p levels differentiated cancer patients from high-risk controls, and, interestingly, might predict disease relapse and response to tyrosine kinase inhibitor [13]. 

Lung cancer screening has been related not only to extravesicular miRNA content but also to EV features. In a preliminary study on 39 serum samples, liquid chromatography–mass spectrometry, Western blot, and immunohistochemistry were sequentially applied to detect potential serum exosomal proteins with altered expression among patients with advanced lung adenocarcinoma, early lung adenocarcinoma, and healthy controls [14]. Among the 672 proteins identified, the integrin alpha M chain (ITGAM) and the glycoprotein clusterin were considered potential markers of lung adenocarcinoma. 

The targeted mass-spectrometry-based approach enabled the detection of 352 lipids in serum EV from lung cancer patients and controls but the high heterogeneity of the observed lipid profiles markedly impaired classification models based on specific compounds [15]. Of interest, the number of small EV and protein levels were significantly increased in the plasma of NSCLC patients compared to healthy donors [16]. Other proteomic studies suggest that circulating EV may encompass specific proteins that might represent biomarkers of lung cancer [17,18,19,20]. Among the identified proteins, extravesicular factor XIII A and complement factor H-related protein 4 were dysregulated in SCLC patients [17]. The exploratory analysis of the proteomic profile of EV content in NSCLC patients who responded well to immune check point compared to non-responders showed differential expression of Annexin A2 and S100A9, proteins involved in immune escape regulation in cancer [18]. The culture of cisplatin-responsive cancer cells was characterized by differentially expressed EV. Such heterogeneity was attributed to the disturbed transcriptional regulation in parental cancer cells [19]. MALDI-TOF mass spectrometry revealed the upregulation of 55 proteins in the serum of patients with cancer compared to healthy controls. Among these, the expression of CD5L only, also known as apoptosis inhibitor 6, is related to that in cancer tissue suggesting its potential application as a lung cancer biomarker [20].

Moreover, a few studies reported lung cancer signatures based on long non-coding RNAs [21,22] or circular RNAs [23]. 

Beyond lung cancer screening and toward its diagnosis, the EV ability to share original cell proteins might also be applied to anticipate, by the analysis of EV in body fluids, the molecular or immunological profile of lung cancer, conventionally provided by bioptic samples. Once optimized on cell culture [24], tissue PDL-1 status was effectively predicted by PDL-1 expression level on circulating EV [24,25]. Moreover, another study monitored the longitudinal change in extravesicular PDL-1 before and after 8 weeks of anti-PD1 treatment and showed that the levels increased in non-responders and decreased in responders [26]. 

In the last two decades, the concept of liquid biopsy has been increasingly expanded. Initially, it referred to the analysis of circulating tumor cells performed to provide information about solid tumors. Lately, it expanded to encompass several tumor-derived markers such as circulating tumor (ct)DNA (the most widely applied one), circulating cell-free RNA, and EV [27]. Especially in specific settings, exosomes can offer a powerful alternative to tissue biopsy and conventional ctDNA isolation. Reclusa et al. demonstrated that the patients’ exosomes can provide a sensible and specific option to detect EML4-ALK translocation in NSCLC [28]. Exosomal analysis represents a minimally invasive procedure, relatively easy to perform and with an affordable cost that might be key to improving the identification of rare mutations/translocations in oncogenes in the future.

## 3. Extracellular Vesicles in Body Fluids and Lung Cancer

**Figure 1:**
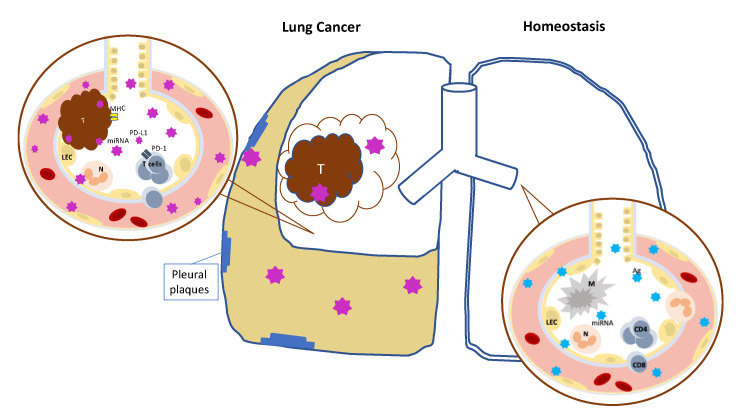
Extracellular vesicles (EV) are released by every cell type. In homeostasis (on the right), EV (in light blue) realize the cross talk between lung epithelial cells (LEC) and immune cells (including macrophages (M), neutrophils (N), lymphocytes (CD4 and CD8)). When lung cancer develops, it produces EV that, in cooperation with parallel mechanisms, can favor immune escape and promote metastasis. Tumor (T)-derived EV (in purple on the left) can be detected in pleural and bronchoalveolar lavage fluid, providing useful samples to study their content in terms of micro RNA (miRNA), major histocompatibility complex (MHC), and check point ligands (PD-L1).

A case could be made that the analysis of body fluids directly in contact with the affected organ—bronchoalveolar lavage fluid and pleural effusion in lung cancer—might offer a more reliable source for the study of the disease (Figure 1). 

### 3.1. Extracellular Vesicles in Airway Samples 

Comparing NSCLC patients with and without EGFR mutation, Hur et al. tested the performance of plasma EV–DNA finding a 55% sensitivity. Looking for more powerful biomarkers, they focused on EGFR genotyping using BAL fluid EV DNA and found 100% accordance with tissue typing [29]. Kim et al. assessed EGFR mutation on BAL EV from 224 stage III-IV NSCLC patients and compared it with tissue genotyping and plasma liquid biopsies. They found a 97% concordance between BAL EV genotyping and standard tissue-based genotyping, much higher than that of plasma cfDNA-based liquid biopsy (63.6%). Plasma-based genotyping performed well in patients with extended disease (sensitivity 40–62%), whereas its sensitivity significantly decreased in limited intrathoracic disease (19–33%). On the other hand, BAL-based EGFR genotyping showed nearly 100% sensitivity regardless of cancer extension. Another advantage of BAL EV typing is that the mean turn-around time of BAL was found to be significantly shorter (2.6 days) than that of tissue-based genotyping (13.9 days; *p* < 0.001) [30]. Moreover, the assessment of BAL EV DNA offers a promising source for identifying acquired resistance in patients on EGFR TKIs. Such resistance usually results from secondary EGFR mutation and its identification relies on new histological samples. pT790M, one of the most common resistance mutations, was successfully detected in BAL EV from NSCLC patients [29].

Another group chose to analyze bronchial washing, a bit less invasive than BAL, finding an overall EGFR mutation detection sensitivity of 89.7% [31]. 

All these results suggest that EGFR genotyping on the EV in airway samples is a promising and optimized tool that might soon replace tissue biopsy in selected candidates. 

Similarly to blood, the content of BAL EV has been the object of studies looking for the best diagnostic and prognostic predictors in lung cancer. The expression levels of two miRNAs, miR-126 and Let-7a, were significantly increased in BAL EV of patients with adenocarcinoma compared to healthy controls. Moreover, these miRNAs were detected in patients at an early stage, suggesting that they might be applied as sensitive markers even when the burden of disease is low and the tumor is most curable. In addition, when lung tissue was assessed, an increased expression of miR-126 in patients with adenocarcinoma compared to controls was still observed, supporting the hypothesis that the EV miRNA signature may be a representative sample of the tumor microenvironment [32]. Proteomic analysis, based on liquid chromatography–mass spectroscopy, of BAL from lung cancer and noncancer patients showed that EV are the most protein-enriched cellular component (compared with cytosol, nucleus, extracellular space, and plasma membrane). Moreover, 35% of the proteins, with significantly different levels between lung cancer and control patients, were classified as EV proteins [33], another hint of their peculiar performance as cancer biomarkers. Among the 133 differentially expressed proteins, metabolic enzymes and proteins of the chaperonin complex were significantly upregulated among lung cancer patients (Table 1). Further insights into airway fluid EV as tumor sensors and/or their pathogenic role are going to be revealed by proteome investigations. Carvalho et al. compared BAL EV in lung cancer patients and controls and observed a significant upregulation of proteins involved in the ERK and nicotinamide adenine dinucleotide phosphate (NADP) binding pathways in the tumor group [34]. Tumor cells have sustained levels of NADPH which is critical for redox control, cell proliferation, and senescence prevention [35]. The protein DNA (cytosine-5)-methyltransferase 3β (DNMT3B) complex, as well, was significantly upregulated in BAL EV from lung cancer patients [34]. Aberrant transcripts of DNMT3B, found in solid cancers, are able to inactivate tumor suppressor genes and thus represent possible therapeutic targets [34]. Moreover, proteome assessment on BAL EV highlighted a majority of B-cell-related markers, an interesting finding since BAL composition is mainly composed of macrophages (60%). Intratumoral B cells have been isolated in lung cancer tissue and might be responsible for antibody production that modulates tumor-reactive T lymphocytes [34]. Collectively, such results suggest that BAL EV are far from being simple cell trash and that, more likely, they are actively interacting in tumor development, offering a reliable mirror of tissue pathology and possible powerful targets for therapies. 

Nonetheless, as outlined by the guidelines [5], the study of EV is complex, multifaceted, and largely anarchic since methods are seldom replicable among different investigators. Indeed, the results provided by different investigation techniques are far from consistent and might require further optimization. When challenged for comprehensive molecular profiling in a clinical next-generation sequencing (NGS) panel, EV DNA from BAL showed significant limitations related to the low tumor purity which resulted in the reduction of effective coverage of variant alleles in tumor cells [36]. As a matter of fact, BAL EV do not include only tumor-cell-derived vesicles, but also those from immune and epithelial cells and extravesicular DNA is comprehensibly a mix of these cell types. Thus, NGS analysis on BAL EV is limited and requires higher sequencing coverage than tissue. Nonetheless, with a specific issue in mind, it can provide peculiar data on tumor molecular profiles. Indeed, the mutation concordance between tissue and BAL EV DNA was not satisfying (56%) but increased to a promising 81% focusing on clinically significant mutations. Moreover, BAL EV DNA performed very well as a score of “tumor mutation burden“ compared to tissue (Pearson’s correlation 0.64, *p* < 0.001) suggesting that BAL EV-based liquid biopsy might be a powerful predictor of immunotherapy response. 

Overall, airway EV represent a promising source for diagnostic and prognostic purposes in lung cancer patients even though data interpretation requires a critical evaluation of the applied investigational methods. In the next future technical standardization and optimization are demanded in order to proclaim the application of BAL EV into routine oncological practice. 

### 3.2. Extracellular Vesicles in Pleural Fluid

Pleural effusion contains a great variety of cells, and among them, we can identify mesothelial cells, macrophages, lymphocytes, leukocytes, as well as tumor cells when a malignant condition is present [37].

In lung cancer, the tumor microenvironment is a dynamic regulator of tumor progression and metastasis development. In this regulation, the formation of EV is a way to transfer signals and permit the cell-to-cell interaction between malignant cells or between cancer and noncancer cells in lung tissue as well as in pleural effusion, playing a critical role in cancer progression.

The first study that reported the isolation of exosomes in malignant effusion was performed on the ascitic fluid in 2002 by Andre et al. [38]. Besides their recognition of the exosomes, they also showed the immunogenic role of tumor-derived exosomes by inducing differentiation and expansion of tumor-specific cytotoxic T cells from peripheral blood cells.

Then, a few years later, exosomes were identified even on malignant pleural effusion [39,40] caused by mesothelioma or metastatic involvement (lung, breast, and ovarian cancer). The most common proteins identified in the exosome fraction from the malignant pleural effusions were immunoglobulin peptides, as well as proteins involved in antigen presentation, signal transduction, migration, and adhesion (e.g., Sorting-nexin family, Thrombospondin 2, pigment epithelium-derived factor, and annexins).

An attempt to better characterize EV in malignant pleural effusion was performed by Park and colleagues [41] using a global proteomic analysis, in non-small-cell lung cancer (NSCLC) pleural involvement. They identified EGFR as a specific pleural effusion exosomes protein of NSCLC, as well as other EGFR interacting proteins (e.g., Grb2, calmodulin, CD59, and Rab5), which is in line with similar findings in various types of human body fluids including colorectal cancer ascites and urine [42,43]. Although the precise functional role of the identified exosomal proteins was not precisely established, many of the isolated proteins may reflect pathological processes, such as tumorigenesis, that could become a possible diagnostic or prognostic biomarker of NSCLC. These studies are limited by the small sample size and by the absence of controls but suggest that pleural effusion EV can provide a novel perspective on cancer with pleural involvement.

In this regard, in a recent study [44], EV isolated from a pleural effusion (*n* = 27) caused by pleural mesothelioma, metastatic adenocarcinoma, and benign mesothelial proliferations, were studied to evaluate their possible diagnostic role. This study showed the presence of 15 surface proteins that regulate a variety of cellular processes including cell–cell adhesion (CD2, CD8, CD9, and CD146), extracellular matrix regulation (CD44), immune regulation (CD24, CD40), TGF-β receptor activation (CD105), as well as cell growth, differentiation, and migration that were higher on the EV fraction derived from pleural effusion of metastatic adenocarcinoma compared to malignant pleural mesothelioma. By contrast, HLA-DRDPDQ and ROR1 were reduced in metastatic adenocarcinoma compared to malignant pleural mesothelioma. This suggests the presence of specific regulatory signals that differentiate the pathogenetic mechanisms of the primary tumor and the metastatic involvement of the pleura. Additionally, Galectin-1, Mesothelin, Osteopontin, and VEGF were higher in EV derived from malignant pleural mesothelioma compared to those with benign disease.

Similar results were reported by Luo et al. [45] in a comprehensive analysis of metabolomics and lipidomics, which investigated the characteristics of two EV subpopulations (large and small) derived from pleural effusion, to assess whether the metabolites could differentiate malignant pleural effusion from pleural tuberculosis. In pleural large EV, a panel of four biomarker candidates, including phenylalanine, leucine, phosphatidylcholine 35:0, and sphingomyelin 44:3, showed high performance for distinguishing pleural tuberculosis and malignant pleural effusion, particularly in patients with a delayed or missed diagnosis. Indeed, the current clinical diagnosis of tubercular and malignant pleural effusion still represents a clinical challenge, particularly for patients with a low load of *Mycobacterium tuberculosis* or tumor cells in the pleural effusions, and especially in those who are too frail to undergo thoracoscopy [46].

**Table 1:** Extracellular vesicles in lung fluids: original articles on lung cancer patients.

Source	EV Study Technique	Detected EV Cargo	Main Message	Year	Ref
BAL	Immuno-electron microscopy; EGFR genotyping	EGFR mutation p.T790 mutation in acquired resistance to EGFR-TKIs	EGFR genotyping on EV DNA from BAL shows 100% accordance with tissue typing.	2018	[29]
	Centrifugation; EGFR genotyping by PANAMutyper™ R EGFR kit (Panagene, Daejeon, Korea)	47 hotspot mutations in EGFR exons 18–21	97% concordance between BAL EV genotyping and standard tissue-based genotyping; BAL EV turn-around time shorter than that of tissue-based genotyping.	2022	[30]
	Centrifugation; miRNA Isolation Kit; reverse transcription + quantitative PCR	miR-7, miR-17, miR-9, miR-21, miR-126, miR-Let-7a	Increase in miR-126 and Let-7a in BAL EV from adenocarcinoma patients compared to controls, both detectable in early stages.	2018	[32]
	Centrifugation; LC-MS	133 differentially expressed proteins in lung cancer and controls. Upregulated: metabolic enzymes and CCT chaperonin complex	BAL proteome-based diagnostics can provide a base for stratifying lung cancer risk.	2017	[33]
	Ultracentrifugation; flow cytometry; NTA; LC-MS analysis; TEM	Upregulated proteins in BAL and tissue from patients with lung cancer: ERK pathways, NADP binding, DNMT3B. Downregulated: ECM composition.	Deep protein analysis can identify cancer signatures.	2020	[34]
	Ultracentrifugation; TEM; EGFR genotyping; NGS	BAL EV DNA and tissue DNA	Low tumor purity of BAL EV DNA compared to tissue DNA.	2021	[36]
Bronchial washing	Exosome identification kit; EGFR genotyping	EGFR mutations: L858R, 19del and T790M	EGFR mutation detection sensitivity of 89.7%.	2020	[31]
Pleural fluid	Ultracentrifugation; electron microscopy; MALDI-TOF mass spectrometry; Western blotting	Immunoglobulin light and heavy chain (G, M) and Ig kappa light chain; complement factors (C1q, C1r, C4a, H), actin; MHC class I and II, sorting nexin protein, B-cell translocation gene 1 protein, pigment epithelium-derived factor, basement membrane-chondroitin sulfate proteoglycan protein, thrombospondin-2	Immunoglobulin peptides as well as proteins involved in antigen presentation, signal transduction, migration, and adhesion were the most common proteins identified in the exosome-containing fractions from the malignant pleural effusions.	2004	[39]
	Ultracentrifugation; EM; LC-MS; proteomic database comparison	264 proteins derived from lung tissue, 67/264 common in lung cancer. Mostly involved in signal transduction (16%), intracellular protein traffic (10%), and immunity (8%).	Signal transduction proteins are prevalent in NSCLC pleural fluid. Among these, EGFR pathway is especially enriched.	2013	[41]
	Centrifugation; NTA; western blotting	Osteopontin, Galectin-1, Mesothelin, and VEGF are higher in mesothelioma. 15 surface proteins (CD9, CD63, CD81, CD2, CD8, CD14, CD29, CD44, CD49e, CD62p, CD105, CD146, CD326, HLA-ABC, and MCSP) were higher in adenocarcinoma. Angiopoietin-1 higher in benign samples.	Relevant EV markers are differentially expressed in malign and benign effusion.	2021	[44]
	Ultracentrifugation; NTA; TEM; western blotting; LC-MS based metabolomic and lipidomic profiling	579 metabolites identified. Malignant EV carry fewer aminoacids, acylcarnitines, phosphatidylcoline, and sphingomyelin than EV in pleural tuberculosis.	Large and small EV vehicles have different metabolites and their cargo differs among tubercular and malignant pleural fluid.	2020	[45]

BAL: bronchoalveolar lavage; EV: extracellular vesicles; TKI: tyrosin kinase inhibitors; NADP: nicotinamide adenine dinucleotide phosphate; LC-MS: liquid chromatography–mass spectrometry; TEM: transmission electron microscopy; NGS: next-generation sequencing; NTA: nanoparticle tracking analysis; ECM: extracellular matrix; MALDI-TOF: matrix-assisted laser desorption/ionization–time of flight.

Main findings are summarized in Table 1.

## 4. EV in Lung Cancer Pathogenesis

The claimed role of EV as biomarkers in lung cancer screening and prognosis is based on the assumption that they are released and accumulate in this condition. The following paragraph is aimed to elucidate whether EV production is a consequence or a trigger of lung cancer.

Several in vitro studies have shown that oxidative stress and inflammation can modify EV secretion from parental cells promoting pathological changes in EV signaling [47,48]. Human airway cells exposed to cigarette smoke showed significant changes in the EV content of miRNAs [49,50]. Focusing on miRNA possibly involved in carcinogenesis, Heliot et al. found significant downregulation of let-7e, let–7 g, and miR-26b in BAL EV from smokers compared to those from non-smokers [51]. Reduced expression levels of miRNAs from the let-7 family are linked to poor outcomes in lung, ovarian, gastric cancer, and hepatocellular carcinoma [52]. The let-7e and let–7 g reduction in EVs from smokers versus non-smoking controls might represent an early response to smoke exposure and might be implicated in later transformation of epithelial cells. On the same line, miR-26b expression was found to be downregulated in NSCLC tissues (N = 154) compared to normal specimens (N = 63) and declined with the survival rate in NSCLC at the median 40 months follow-up (N = 133). Such results indicate that miR-26b might be useful in both the diagnostic and prognostic processes of NSCLC patients [53]. The comparison of EV populations in the lungs of smokers with NSCLC and control smokers and non-smokers showed that EGFR, KRAS, ALK, MET, LKB1, PIK3CA, and ROS1, proteins encoded by genes that are often associated with NSCLC, were overexpressed in lung exosome from smokers and NSCLC patients [54]. These results suggest that smoking induces a specific EV release in the lungs of smokers that might anticipate and favor tumorigenesis.

EV can be crucial, as well, in the pathogenetic mechanism of lung cancer due to professional exposure to hazards. Asbestos exposure causes specific changes in the proteomic cargo of the exosomes secreted from lung epithelial cells and macrophages [55]. Furthermore, such asbestos-induced exosomes promoted gene expression changes related to epithelial-to-mesenchymal transition and other cancer-related genes in human mesothelial cells [55]. Animal and cell models suggest that EV might actively participate in the tumorigenesis induced by pollution. Wang and colleagues found that chronic PM2.5 exposure causes bronchial epithelial cells atypical hyperplasia and induces epithelial-to-mesenchymal transition in vivo, and identified a differential expression of miRNA exosomes in bronchial cell culture [56] providing a base for future studies in vivo.

A crucial step in carcinogenesis is the failure of immune surveillance. Indeed, the tumor is characterized by neoantigens that initially trigger the host immune response. With disease progression, the tumor escapes from self-protection mechanisms, especially by the establishment of an immunosuppressive state within the tumor microenvironment [57]. EV released by lung tumor cells or immune cells convey several bioactive molecules, such as proteins, lipids, and nucleic acids, which are shared between cells and can modulate immune processes [58]. In most cases, EV from lung tumor cells shield substances that can favor immune escape [59,60]. The final effect of EV depends on the tumor environment since both immunosuppressive and immunostimulatory cargo have been described as collectively reviewed by Yin et al. [61]. Even though in a model different from lung cancer, Plebanek et al. observed that exosomes from poorly metastatic melanoma cells can limit cancer metastasis to the lung by stimulating an innate immune reaction and triggering cancer cell clearance at the pre-metastatic niche [62]. By contrast, exosomes from advanced and highly metastatic melanoma were shown to promote the creation of pre-metastatic niches in remote microenvironments to favor metastasis [63]. A potential future approach to lung cancer will address the mechanisms by which EVs mediate lung cancer immunity. Further evidence supports the crucial role of EV in cancer spread. Indeed, EV are able to promote the proliferation of lung cancer cells, inhibit apoptosis, and regulate the invasion and migration potential [64]. Wnt3a/β-catenin [65], circSATB2 [66], HIF-1α/COX-2 [67], and LMO7 [68] in tumor-derived EV modulate genes or signaling pathways promoting the successful proliferation and migration of tumor cells. Moreover, the extravesicular miRNAs miR-326, miR-135b, miR-210 [67], miR-660-5p, and miR-96 [68] vehiculated by tumor cells were shown to favor cancer progression and metastatic spread. Hypoxia stimulus, recreated in culture, can promote protein synthesis and the release of specific EV by tumor cells that might promote inflammation, angiogenesis, and, therefore, tumor progression [69]. Tumor-derived EV released by lung cancer cells regulate the expression levels of genes encoding the matrix metalloproteinases MMP-2, MMP-9, PTEN, E-cadherin, and ROCK1, crucial in the regulation of cell-matrix composition [64]. By promoting epithelial–mesenchymal transition of tumor cells and inducing angiogenesis, the EV released by lung cancer cells can establish a pre-metastatic microenvironment, favor immune escape and guide the formation of a metastatic niche [70].

Moreover, increasing evidence supports the role of EV in cancer therapeutic resistance. Glioblastoma tumor cells can internalize bevacizumab, the only anti-angiogenic agent approved for selected NSCLC patients, and then release it as EV. The inhibition of such EV-mediated drug entrapment increases the bevacizumab anti-tumor activity in vitro [71]. The presence of carcinoma-associated fibroblasts in the tumor stroma favors cancer progression by releasing EV that create the pre-metastatic niche [72] and EV that convey long non-coding RNA small nucleolar RNA host gene 12 (LncRNA SNHG12), responsible for cisplatin resistance in NSCLC [73] and doxorubicin resistance in osteosarcoma [74]. EV seems to be involved, as well, in the acquired resistance to osimertinib, the tyrosine kinase inhibitor that represents the first line treatment for patients with advanced NSCLC harboring EGFR mutation. The long non-coding RNA MSTRG.292666.16 vehiculated by exosomes can provide osimertinib resistance in NSCLC [75]. According to Wu et al., the EV released by EGFR wild type NSCLC cells promote osimertinib resistance in NSCLC cells carrying EGFR mutation [76], suggesting that tumor heterogeneity is critical in treatment refractoriness. The intriguing results of such studies, based on cell culture and in vitro experiments, require further evaluation before being translated into successful clinical applications.

## 5. Conclusions

In the last decades, several discoveries in the pathogenesis of lung cancer have led to the development of new targeted therapies and the identification of the central role of the host immune system and response. Moreover, there is accumulating evidence that EV take part in tumor development and progression suggesting their valuable application as lung cancer diagnostic and prognostic biomarkers.

Indeed, the presented studies collectively support that EV are far from being “dusty cellular trash”, representing instead effective conveyors of crucial messages in lung cancer patients. Nonetheless, there is a methodological heterogeneity that can lead to nebulous results and deserves critical evaluation before using these particles in daily clinical practice. As predictable, the best way to investigate circulating EV in tumor patients is by assessing their content: very peculiar miRNA signatures and protein expression have been identified even in early lung cancer, suggesting their potential application as sentinels in cancer screening. Nonetheless, the messages conveyed by peripheral EV can be blurred by concomitant morbidities or acute events that can limit their application.

In contrast, the study of airway EV, which are directly in contact with the affected organ, offers a strict reflection of lung events in tumor pathogenesis, with fewer influencing factors. Indeed, EGFR genotyping in BAL EV seems extremely promising and might, in the future, be preferred to tissue sampling.

Interestingly, cancer resistance to treatment (TKI, antiangiogenic agents, and chemotherapies) could soon be predicted by the assessment of the EV released by cancer cells and the surrounding microenvironment.

In addition, by capturing the signaling between tumor and non-tumor cells floating in BAL and in pleural fluid, we might obtain deeper insights into the pathogenic mechanisms leading to lung cancer development and progression.

Finally, by further optimizing the assessment and our understanding of lung EV, we might bridge the growing gap between the need for exhaustive molecular and immunological tumor features and the purpose of performing less invasive diagnostic procedures on fragile patients.

## Data Availability

Not applicable.
